# Improving the Quality of Diagnostic Studies Evaluating Point of Care Tests for Acute HIV Infections: Problems and Recommendations

**DOI:** 10.3390/diagnostics7010013

**Published:** 2017-03-04

**Authors:** Megan Smallwood, Nitika Pant Pai

**Affiliations:** Division of Clinical Epidemiology & Infectious Diseases, Department of Medicine, McGill University, Montreal, QC H3A 0G4, Canada; megan.smallwood@mail.mcgill.ca

**Keywords:** quality, diagnostics, acute HIV infection, recommendations, point of care

## Abstract

The diagnosis of acute human immunodeficiency virus (HIV) infection (AHI) plays a unique role in preventing the spread of HIV and ending the epidemic. Acutely infected individuals are thought to contribute substantially to forward transmissions of HIV; however, diagnosing AHI in resource-limited settings has proven to be a challenge. While fourth generation antigen-antibody combination assays have been successful in high-resource settings, rapid point of care (POC) versions of these assays have yet to demonstrate high sensitivity to detect AHI. Newer RNA/DNA based POC technologies are being validated, but the challenge to understand the additional value of these devices depends on the quality of study evaluations, in particular choice of study designs and case mix of included populations. In this commentary, we aimed to review the quality of studies evaluating a new fourth generation rapid test for detecting AHI, to identify general methodological limitations and biases in diagnostic accuracy studies, and to recommend strategies for avoiding them in future evaluations. The new studies that were evaluated continued to report the same weaknesses and biases that were seen in previous evaluations of fourth generation rapid tests. We recommend that investigators design future studies carefully, keeping in mind how diagnostic performance may be influenced by prevalence, population, patient case mixes, and reference standards. Care must be taken to avoid biases specific to diagnostic accuracy studies (spectrum, verification, incorporation and reference standard biases). To improve on quality, reporting checklists and guidelines such as Quality Assessment of Diagnostic Accuracy Studies (QUADAS-2) and Standards for Reporting Diagnostic accuracy studies (STARD) should be reviewed prior to conducting studies.

## 1. Why Study Acute HIV Infection?

In light of the 90-90-90 United Nations Joint Program on HIV/AIDS (UNAIDS) targets, identifying the estimated 19 million human immunodeficiency virus (HIV) positive individuals worldwide who are unaware of their HIV status remains a priority [1,2]. Early detection of HIV infection is key to saving public health costs [3], and diagnosis of acute HIV infection (AHI) is gaining global attention. Acute infection is generally defined as the period of infection which precedes seroconversion, when RNA is detectable in the blood but antibodies are not [4]. AHI is thought to contribute disproportionately to new infections, with modeling estimates suggesting that acute and early HIV infection account for up to 50% of all forward transmissions [5,6]. The benefits to detecting HIV during early infection include earlier treatment initiation (now WHO recommended), resulting in reductions in viral load [7]. Detection during AHI has public health implications for reducing forward transmission of HIV, in addition to management of symptoms of acute retroviral syndrome and lowering the viral set point [8]. The additional value of detecting AHI must be taken into consideration, for ensuring timely treatment and controlling the spread of HIV.

## 2. Problems Encountered in Detecting AHI

Compared to detecting established HIV (post-seroconversion), there are many challenges unique to the detection of acute HIV infection. Due to the lack of detectable HIV antibodies in the blood during AHI, many diagnostic technologies are rendered ineffective. This is of particular importance for high prevalence resource-limited settings, which rely heavily on rapid tests to detect antibodies to HIV. A recent study in South Africa demonstrated that a substantial number of individuals in the acute and early stage of HIV infection are misdiagnosed as negative upon initial screening with rapid antibody point of care tests [9], highlighting the need for rapid tests which can detect HIV earlier, preferably in outreach settings.

Efforts to detect AHI have so far focused on identifying HIV RNA or p24 antigen, both of which appear in the blood prior to HIV antibodies. RNA is detectable even earlier than p24 antigen (5–7 days earlier), thus nucleic acid amplification testing (NAAT) is still the most sensitive method of detecting AHI [10]. However, NAAT is not practical technology for many settings as it is expensive, and generally requires complex laboratory equipment and skilled technicians to perform.

Early infant diagnosis (EID) faces similar challenges for detecting acute HIV, and a point of care NAAT (Alere™ q HIV 1/2 Detect) has been developed for the purpose of EID [11]. This device may have potential to detect AHI at point of care, yet its performance is currently being reviewed. Field evaluations in a broad spectrum of patients are needed to determine its accuracy, effectiveness and implementation potential for AHI.

Fourth generation laboratory assays detect p24 antigen in addition to HIV antibodies. These assays are highly accurate and less expensive than NAAT, but have encountered barriers to being developed in a rapid, point of care format.

Detection of p24 antigen is challenging due to is its tendency to form immune complexes with antibodies in the blood, a barrier which has been largely overcome with the use of ultrasensitive heat dissociation techniques; however these techniques are difficult to implement into a rapid, point of care diagnostic test prototype [12,13]. Difficulties in sensitive detection of p24 antigen have been seen with the first FDA-approved fourth generation rapid test: the Determine HIV 1/2 Ag/Ab Combo test (Alere) [14].

Our previous work evaluated the overall performance of the Determine HIV Combo rapid test (Alere) in a systematic review and meta-analysis [15]. We found that while the Determine HIV Combo test’s sensitivity to detect HIV antibodies was impressive, it was not able to detect p24 antigen with high sensitivity (thus unable to diagnose AHI) [15]. These findings were largely congruent with a similar review, which investigated rapid fourth generation tests in field evaluations [16]. While the accuracy of the Determine HIV Combo test for detecting AHI was disappointing, our quality assessment of studies highlighted the numerous challenges for conducting high quality diagnostic evaluations of rapid fourth generation tests.

To follow up on our prior meta-analysis, we aim to describe weaknesses in past evaluations of fourth generation rapid tests, evaluate if improvements have been made in recent studies, and address what is needed to improve future evaluations of rapid diagnostic tests for AHI.

### 2.1. Weaknesses and Biases Observed in Study Evaluations

We identified numerous weaknesses and biases in study design and conduct, that were recurring across a total of 17 studies evaluating the Determine HIV Combo test published over a period of four years (2010–2014) [15]. Many of these problems are known to span diagnostic accuracy studies in the field of HIV and sexually transmitted infections. These problems include failure to perform sample size calculations for a defined primary end point, poor selection of study population, and biases such as verification and incorporation biases (especially lack of blinding, and use of complex reference testing algorithms).

The most frequent sources of bias in the evaluation of the Determine HIV Combo rapid test, as described in our meta-analysis and QUADAS-2 (Quality Assessment of Diagnostic Accuracy Studies) summary [17], were risk of biases in patient selection (i.e., case-mix due to case-control study design) and interpretation of the index test due to lack of blinding. While the former poses specific challenges in the context of fourth generation evaluations, the interpreter of an index test in any diagnostic evaluation should always be blinded to the reference standard results. Reviewer bias (due to lack of blinding) is more common in case control studies, but it can also occur in cross-sectional studies depending on the study procedure and time frame (i.e., if the reference standard is conducted before the index test). While the Determine Combo rapid test was designed to be simple to interpret, there is still some subjectivity in its interpretation and blinding should be reported in any diagnostic evaluation.

We have outlined below the various biases and limitations in study design which have specific implications in evaluations for fourth generation combination (antigen/antibody) tests.

### 2.2. Impact of Spectrum Bias in Selecting a Study Design

Spectrum bias has the potential to have a severe impact on accuracy estimates in a study. This bias can occur in case-control studies when patients or blood samples are selected based on HIV status, resulting in a case mix of patients/samples that is not representative of the target population. Patients/samples included in this type of analysis are often at extremes of the disease spectrum (i.e., well characterized and clearly positive or negative), which tends to over-estimate test accuracy. This is critical to keep in mind when evaluating fourth generation combination tests (e.g., the Determine HIV Combo rapid test), which can detect markers for HIV at different stages: p24 antigen and HIV antibody.

An individual who obtains a positive band for p24 antigen, HIV antibody, or both, is considered HIV positive. An individual who obtains a negative result for both p24 antigen and HIV antibody is considered HIV negative. Calculating an “overall” accuracy estimate for this kind of test, therefore, is actually an average of the performance of the two components in a study population, weighted to the proportion of individuals/samples which are antigen or antibody positive. Investigators must therefore be careful of the case mix of patients they recruit, if the sensitivities of the antigen and antibody components are different.

Spectrum bias was observed in the evaluations of Determine HIV Combo rapid test, which has a highly sensitive antibody component but low sensitivity for the antigen component and was evaluated in populations with varying case mixes. Using the evaluations of this test as an example, we noted that cross-sectional studies tended to have a higher proportion of patients in the established/post-seroconversion stage than patients in the acute phase (due to the narrow time frame for detecting AHI), resulting in a higher overall sensitivity. In order to increase the sample size to evaluate the p24 antigen component, many case-control studies specifically sought out p24 antigen positive samples, which naturally overestimates the prevalence of p24 antigen that would be found even in the highest prevalence setting, precipitating spectrum bias. In these cases, the overall performance of the Determine HIV Combo rapid test was more heavily weighted to the antigen component of the test (compared to field evaluations), resulting in a lower overall sensitivity.

Spectrum bias can be avoided with careful sampling, to ensure that patients/samples represent the full spectrum of disease that would be expected in a population. When previously stored blood/plasma specimens are included, randomizing the selection of these samples can mitigate this risk. In an observational cross-sectional study, a consecutive or random sampling strategy should be considered.

### 2.3. Impact of Imperfect Reference Standard Bias on Accuracy Estimates

Selecting an appropriate reference standard for evaluating the accuracy of fourth generation tests such as the Determine HIV Combo test is particularly challenging, as the Determine Combo test provides independent results for p24 antigen and HIV antibody. The various assays used as reference standards (or as part of reference testing algorithms) for the Determine Combo test were third and fourth generation enzyme immunoassays (EIA), third generation rapid tests, NAAT, Western Blot, and p24 antigen assays. If the accuracy of each test component of the Determine Combo is to be evaluated separately (as was the case), then an appropriate reference standard must be selected which can accurately identify the true presence or absence of p24 antigen or HIV antibody, independently. For example, using a third generation test as the sole reference standard is only suitable when evaluating the performance of the antibody component of a fourth generation test, by nature they cannot detect p24 antigen. This was the case for the evaluation conducted by Bhowan and colleagues, where the reference standard algorithm consisted of two third generation rapid tests in parallel [18]. A fourth generation assay was only used in cases where the reference tests were discordant, or the Determine Combo rapid test was discordant with the third generation tests. As such, only the antibody component of the test could be evaluated—the investigators were not able to evaluate the overall performance of the assay, nor its ability to detect acute infections. Additionally, NAAT should be used with caution as a reference standard, as there is a 5-day window when RNA is detectable but p24 is not, meaning sensitivity for the p24 antigen component will be underestimated. For example, in the study by Patel et al. [19], the Determine Combo rapid test was compared to NAAT, likely estimating a lower overall sensitivity than had it been compared to a fourth generation assay, as there may have been infections that were NAAT positive but not yet p24 antigen positive. Nevertheless, it may be acceptable to use NAAT as a reference standard if the objective of the study is to evaluate Determine Combo test’s sensitivity to detect AHI in general (rather than p24 antigen). Authors should clearly state this distinction in their study objectives, and interpretation of results.

For estimating the overall accuracy, an appropriate reference standard for the Determine HIV Combo test would be a highly accurate fourth generation laboratory assay (e.g., Abbott Architect Combo assay). Conway and colleagues evaluated the Determine Combo rapid test against a fourth generation laboratory assay, with additional supplementary testing for positive specimens (with HIV antibody test, sensitive p24 assay and Western Blot), and samples were classified as acute if they were p24 antigen positive but antibody negative [20] This allowed for proper estimation of overall sensitivity as well as each independent test component (p24 and HIV antibody).

Lastly, the choice of statistical methods also plays a role in evaluating test performance. For example, advanced Bayesian statistical methods can help overcome limitations in Frequentist statistical methods typically used for correcting biases due to imperfect reference standards and incorrect verification [21,22].

Given the biases frequently seen in the studies evaluating the Determine Combo, addressing the question of test suitability for detecting acute HIV becomes complicated. The following questions remain: is the accuracy (e.g., sensitivity, specificity) more likely to be over or under-estimated in light of these biases, and can they feasibly be avoided in studies of diagnostic accuracy?

We ask these questions again in the context of a new fourth generation rapid test for HIV developed by Alere, the Alere HIV Combo test [23] which is anticipated to be an improvement upon the Determine HIV Combo rapid test.

## 3. The Alere HIV Combo Test: A Summary of Evaluations

Since our review of the Determine Combo rapid test, four published studies have evaluated the new Alere HIV Combo test [24,25,26,27]. All four studies are case-control designs. The studies were conducted in Switzerland (this study did not compute accuracy estimates e.g., sensitivity, specificity) [27], United Kingdom [24], Japan [25], and the Netherlands [26]. So far, the Alere HIV Combo test has shown improvements in p24 antigen sensitivity compared to the Determine HIV Combo rapid test [24,25]. Nevertheless, these studies have fallen victim to some of the same design flaws (outlined below) which were identified in the Determine HIV Combo rapid test evaluations.

We critiqued three studies (excluding the full text in Japanese for lack of translation) using the validated QUADAS-2 method [15,17]. Limitations and biases in these studies have been outlined in Table 1.

Risk of bias in patient selection due to the use of a case-control design was expected in the initial evaluations, as there are distinct advantages to evaluating the performance of a novel test using a case-control study, under “ideal” (i.e., laboratory controlled) environment before undertaking field evaluations. Two out of the three studies failed to mention blinding of reference standard results when interpreting the index test [24,27], the third study acknowledged that the interpretation was unblinded (but did not provide any explanation) [26].

The evaluation by Fitzgerald and colleagues restricted their samples to those which were (by reference standard) either HIV negative, p24 antigen positive, or HIV antibody positive, but did not include any samples which were positive for both p24 antigen and HIV antibody by reference standard, putting this evaluation at risk of spectrum bias. This evaluation missed a key group of individuals whose antibodies may not yet be picked up by a second or third generation antibody assay, but could be captured as positive through the p24 antigen component of the Alere HIV Combo test.

### 3.1. Recommendations

High quality studies, especially field evaluations, evaluating the Alere HIV Combo (or any similar fourth generation rapid test) in the future will be essential to diagnosing AHI in low and middle income settings. Quality issues can be avoided during the design stage of research studies. To avoid the biases which plagued evaluations of the Determine HIV Combo rapid test, we propose the following recommendations for future evaluations of Alere HIV Combo.

The use of a cross-sectional study design is recommended. It is essential to understand how a diagnostic test, particularly rapid and point of care tests, will perform outside of a controlled environment, in field settings. The advantages of using cross-sectional designs compared to case-control studies involve avoiding spectrum bias, obtaining generalizable results, and a more realistic indication of how a diagnostic test is likely to perform once it has been implemented in a health care setting. Cross-sectional studies (even with large sample sizes) may face difficulty recruiting sufficient numbers of patients with acute HIV infection to evaluate the performance of fourth generation rapid tests (such as Determine HIV Combo) during AHI [28]. However, a recent study from the US was able to overcome this challenge in identifying acute infections by implementing a more sensitive test algorithm; in this study the Architect Ag/Ab fourth generation assay was used in addition to pooled RNA testing to identify acute infections [29].

In contrast, there are advantages to using a case-control design when initially investigating a novel test or perfecting a prototype, for achieving an adequately large sample size of p24 positive specimens. The short window period of acute HIV detection (2–4 weeks) makes it challenging to capture a sufficient sample size of acute specimens (p24 antigen or RNA positive, but antibody negative). It is understandable as to why many researchers turn to a case-control design over a cross-sectional design, particularly in early evaluations where achieving perfection of device prototype becomes the guiding principle for future studies. Regardless of study design, researchers should make efforts to include all types of samples from participants of varying risk profiles, in different stages of HIV infection, not just those which are very clearly positive.

Finally, STARD is a reporting checklist for diagnostic accuracy studies [30]; QUADAS-2 is a useful tool for understanding how biases can occur due to flaws in study design, and is frequently used to assess study quality when conducting systematic reviews of diagnostic accuracy studies [17]. Both STARD and QUADAS-2 guidelines are practical and can be consulted while designing study protocols so that potential issues in quality are identified ahead of time.

### 3.2. Other Considerations

While we wait for the results of future high quality studies evaluating new tests for acute HIV infection, it is worth mentioning alternative approaches. These approaches are being used to identify acutely infected individuals in resource-limited settings, which may prove useful in the absence of a high performing fourth generation rapid point of care test. One such approach being used in sub-Saharan Africa is targeted screening of individuals, based on signs and symptoms associated with AHI (e.g., symptoms of acute retroviral syndrome such as fever, or receiving discordant rapid HIV test results) [31] This approach involves the creation of a risk score algorithm, where only high-risk patients undergo laboratory testing for AHI, and has been shown to perform well and reduce the number of patients undergoing resource-intensive testing for AHI. Other risk score algorithms may be useful in different populations (including high income settings), such as the Denver risk score which has been validated in previous studies [32].

## 4. Conclusions

To conclude, a highly sensitive fourth generation combo rapid test has the potential to improve the detection of acute infections, however overall study quality (including sampling methodology, study design, statistical methods and reference standard algorithms) needs to be improved in future evaluations. When designing studies to evaluate a novel device, the correct population (case mix) should be targeted to avoid spectrum bias, and investigators should take special care to blind reference standard results when interpreting the index test results. Being mindful of biases and all the key items mentioned above, will not only improve the quality of evaluation, but raise the standard of diagnostic studies for detection of AHI.

## Figures and Tables

**Table 1:** Quality assessment of studies evaluating the Alere human immunodeficiency virus (HIV) Combo test.

Study Authors	Year of Publication	Sample Size	QUADAS-2 Domains *			
			Could the Selection of Patients Have Introduced Bias?	Could the Conduct or Interpretation of the Index Test (Alere HIV Combo) Have Introduced Bias?	Could the Reference Standard, in Its Conduct, or Its Interpretation, Have Introduced Bias?	Could the Patient Flow Have Introduced Bias?
Ottiger et al.	2015	38	high	unclear	low	low
Fitzgerald et al.	2016	120	high	unclear	low	low
Van Tienen et al.	2017	89	high	high	low	low

* Risk: high, low, or unclear.

## References

[1] Joint United Nations Programme on HIV/AIDS (UNAIDS) (2014). The Gap Report.

[2] Joint United Nations Programme on HIV/AIDS (UNAIDS) (2015). Diagnostics Access Initiative to Achieve the 90–90–90 Treatment Target.

[3] Long E.F. (2011). HIV Screening via Fourth-Generation Immunoassay or Nucleic Acid Amplification Test in the United States: A Cost-Effectiveness Analysis. PLoS ONE.

[4] Branson B.M. (2010). The Future of HIV Testing. J. Acquir. Immune Defic. Syndr..

[5] Brenner Bluma G., Roger M., Routy J.P., Moisi D., Ntemgwa M., Matte C., Baril J.G., Thomas R., Rouleau D., Bruneau J. (2007). High Rates of Forward Transmission Events after Acute/Early HIV-1 Infection. J. Infect. Dis..

[6] Wawer M.J., Gray R.H., Sewankambo N.K., Serwadda D., Li X., Laeyendecker O., Kiwanuka N., Kigozi G., Kiddugavu M., Lutalo T. (2005). Rates of HIV-1 transmission per coital act, by stage of HIV-1 infection, in Rakai, Uganda. J. Infect. Dis..

[7] World Health Organization (2015). Guideline on When to Start Antiretroviral Therapy and on Pre-Exposure Prophylaxis for HIV.

[8] O’Brien M., Markowitz M. (2012). Should We Treat Acute HIV Infection?. Curr. HIV/AIDS Rep..

[9] Mayaphi S.H., Martin D.J., Quinn T.C., Laeyendecker O., Olorunju S.A., Tintinger G.R., Stoltz A.C. (2016). Detection of Acute and Early HIV-1 Infections in an HIV Hyper-Endemic Area with Limited Resources. PLoS ONE.

[10] Krajden M., Cook D., Mak A., Chu K., Chahil N., Steinberg M., Rekart M., Gilbert M. (2014). Pooled nucleic acid testing increases the diagnostic yield of acute HIV infections in a high-risk population compared to 3rd and 4th generation HIV enzyme immunoassays. J. Clin. Virol..

[11] Alere (2016). Alere™ q HIV-1/2 Detect. http://www.alerehiv.com/ww/home/hiv-screening/alere-q-hiv-1–2-detect/eid.html.

[12] Nishanian P., Huskins K.R., Stehn S., Detels R., Fahey J.L. (1990). A simple method for improved assay demonstrates that HIV p24 antigen is present as immune complexes in most sera from HIV-infected individuals. J. Infect. Dis..

[13] Schüpbach J., Tomasik Z., Knuchel M., Opravil M., Günthard H.F., Nadal D., Böni J., Swiss HIV Cohort Study, Swiss HIV Mother + Child Cohort Study (2006). Optimized virus disruption improves detection of HIV-1 p24 in particles and uncovers a p24 reactivity in patients with undetectable HIV-1 RNA under long-term HAART. J. Med. Virol..

[14] Alere International Limited (2014). Alere Determine™ HIV-1/2 Ag/Ab Combo. http://alerehiv.com/hiv-screening/features.

[15] Smallwood M., Vijh R., Nauche B., Lebouche B., Joseph L., Pant Pai N. (2016). Evaluation of a Rapid Point of Care Test for Detecting Acute and Established HIV Infection, and Examining the Role of Study Quality on Diagnostic Accuracy: A Bayesian Meta-Analysis. PLoS ONE.

[16] Lewis J.M., Macpherson P., Adams E.R., Ochodo E., Sands A., Taegtmeyer M. (2015). Field accuracy of fourth-generation rapid diagnostic tests for acute HIV-1: A systematic review. AIDS.

[17] Whiting P.F., Rutjes A.W., Westwood M.E., Mallett S., Deeks J.J., Reitsma J.B., Leeflang M.M., Sterne J.A., Bossuyt P.M., QUADAS-2 Group (2011). QUADAS-2: A revised tool for the quality assessment of diagnostic accuracy studies. Ann. Intern. Med..

[18] Bhowan K., Kalk E., Khan S., Sherman G. (2011). Identifying HIV infection in South African women: How does a fourth generation HIV rapid test perform?. Afr. J. Lab. Med..

[19] Patel P., Bennett B., Sullivan T., Parker M.M., Heffelfinger J.D., Sullivan P.S., CDC AHI Study Group (2012). Rapid HIV screening: Missed opportunities for HIV diagnosis and prevention. J. Clin. Virol..

[20] Conway D.P., Holt M., McNulty A., Couldwell D.L., Smith D.E., Davies S.C., Cunningham P., Keen P., Guy R., Sydney Rapid HIV Test Study (2014). Multi-centre evaluation of the Determine HIV Combo assay when used for point of care testing in a high risk clinic-based population. PLoS ONE.

[21] Joseph L., Gyorkos T.W., Coupal L. (1995). Bayesian estimation of disease prevalence and the parameters of diagnostic tests in the absence of a gold standard. Am. J. Epidemiol..

[22] Lu Y., Dendukuri N., Schiller I., Joseph L. (2010). A Bayesian approach to simultaneously adjusting for verification and reference standard bias in diagnostic test studies. Stat. Med..

[23] Alere (2016). Alere™ HIV Combo. http://www.alerehiv.com/ww/home/hiv-screening/alere-hiv-combo/introduction.html.

[24] Fitzgerald N., Cross M., O’Shea S., Fox J. (2016). Diagnosing acute HIV infection at point of care: A retrospective analysis of the sensitivity and specificity of a fourth-generation point-of-care test for detection of HIV core protein p24. Sex. Transm. Infect..

[25] Nakagiri I., Wada H., Tokunaga H., Fukuda H., Tasaka T., Sugihara T. (2015). Performance Assessment of a Newly Developed Rapid Diagnostic Reagent for Human Immunodeficiency Virus. Kansenshogaku Zasshi..

[26] Van Tienen C., Rugebregt S., Scherbeijn S., Gotz H., GeurtsvanKessel C. (2017). The performance of the Alere HIV combo point-of-care test on stored serum samples; useful for detection of early HIV-1 infections?. Sex. Transm. Infect..

[27] Ottiger C., Huber A.R. (2015). Comparison of the New Alere HIV Combo with Alere Determine HIV-1/2 Ag/Ab Combo in Acute Primo and Established HIV Infections. Ann. Clin. Lab. Res..

[28] Duong Y.T., Mavengere Y., Patel H., Moore C., Manjengwa J., Sibandze D., Rasberry C., Mlambo C., Li Z., Emel L. (2014). Poor performance of the determine HIV-1/2 Ag/Ab combo fourth-generation rapid test for detection of acute infections in a National Household Survey in Swaziland. J. Clin. Microbiol..

[29] Peters P.J., Westheimer E., Cohen S., Hightow-Weidman L.B., Moss N., Tsoi B., Hall L., Fann C., Daskalakis D.C., Beagle S. (2016). Screening Yield of HIV Antigen/Antibody Combination and Pooled HIV RNA Testing for Acute HIV Infection in a High-Prevalence Population. JAMA.

[30] Bossuyt P.M., Reitsma J.B., Bruns D.E., Gatsonis C.A., Glasziou P.P., Irwig L., Lijmer J.G., Moher D., Rennie D., de Vet H.C. (2015). STARD 2015: An updated list of essential items for reporting diagnostic accuracy studies. BMJ.

[31] Sanders E.J., Wahome E., Powers K.A., Werner L., Fegan G., Lavreys L., Mapanje C., McClelland R.S., Garrett N., Miller W.C. (2015). Targeted screening of at-risk adults for acute HIV-1 infection in sub-Saharan Africa. AIDS.

[32] Haukoos J.S., Lyons M.S., Lindsell C.J., Hopkins E., Bender B., Rothman R.E., Hsieh Y.-H., MacLaren L.A., Thrun M.W., Sasson C. (2012). Derivation and Validation of the Denver Human Immunodeficiency Virus (HIV) Risk Score for Targeted HIV Screening. Am. J. Epidemiol..

